# n-3 PUFA poor seafood consumption is associated with higher risk of gout, whereas n-3 PUFA rich seafood is not: NHANES 2007–2016

**DOI:** 10.3389/fnut.2023.1075877

**Published:** 2023-04-04

**Authors:** Guixing Zeng, Dongxin You, Lingyan Ye, Yuchi Wu, Hualin Shi, Jiarong Lin, Ziyan Jiang, Junping Wei

**Affiliations:** ^1^Guang’anmen Hospital, China Academy of Chinese Medical Sciences, Beijing, China; ^2^The Second Clinical College, Guangzhou University of Chinese Medicine, Guangzhou, China; ^3^Second Affiliated Hospital, Guangzhou University of Chinese Medicine (Guangdong Provincial Hospital of Chinese Medicine), Guangzhou, China; ^4^Shenzhen Longhua Maternity and Child Healthcare Hospital, Shenzhen, China

**Keywords:** n-3 PUFA poor/rich seafood consumption, gout, inflammation, National Health and Nutrition Examination Survey, nutrition

## Abstract

**Background and aims:**

Gout, the most prevalent inflammatory arthritis, has undesirable effects on the quality of life. Omega-3 polyunsaturated fatty acids (n-3 PUFA) has a strong link with anti-inflammatory impacts. However, whether the harmful effects of seafood in relation to gout may vary owing to different levels of n-3 PUFA in seafood is still unclear. It was the goal of this study to examine the relationship between n-3 PUFA poor/rich seafood consumption and gout.

**Methods:**

Between 2007 and 2016, five NHANES cycles were performed, with 12,505 subjects having complete data for gout and two 24-h dietary intake interviews. The 24-h dietary recalls were utilized to evaluate dietary habits. Gout was defined based on questionnaires. Weighted logistic regression models were conducted to investigate the association between n-3 PUFA poor/rich seafood consumption and gout. Moreover, subgroup analysis was utilized to estimate the stability of results. Covariates including age, gender, race/ethnicity, income, education, body mass index, chronic kidney disease, diabetes mellitus, hypertension, smoking status, and drinking status were stratified in different models.

**Results:**

In the fully adjusted model, each unit of increase of n-3 PUFA poor seafood intake was associated with an 8.7% increased risk of gout (OR = 1.087, 95% CI: 1.039, 1.138, *P* < 0.001), whereas, no correlation was found between n-3 PUFA rich seafood consumption and gout. It also provided a proof-of-concept regarding the potential for n-3 PUFA rich seafood to counteract harmful effects of purines in relation to gout. A dose-response analysis showed that there was a non-linear relationship between n-3 PUFA rich seafood intake and the risk of gout in the female group.

**Conclusion:**

Findings suggest that n-3 PUFA poor seafood consumption is associated with higher risk of gout, whereas n-3 PUFA rich seafood is not.

## Introduction

Gout is an inflammatory crystal arthritis, the most important pathological characteristic of which is monosodium urate deposition in the joints. The incidence of gout has been stably increasing to 0.58–2.89 per 1,000 person-years worldwide with the development of the world economy and lifestyle changes (1). Additionally, gout episodes have undesirable effects on the quality of life (2).

Recent studies have revealed a strong link between omega-3 polyunsaturated fatty acids (n-3 PUFA) and anti-inflammatory impacts (3–5). Numerous systematic reviews and meta-analysis have noted that n-3 PUFAs exhibited anti-inflammatory effects on multiple non-communicable diseases, including systemic inflammatory response syndrome (3), colorectal cancer (4), type 2 diabetic mellitus (5), bipolar disorder (6), mental disorders (7), polycystic ovary syndrome (8), heart failure (9), hypertension (10), and rheumatoid arthritis (11). With respect to gout, experiments *in vitro* noted that n-3 PUFAs could inhibit the NLRP3 inflammasome in monocytes *via* downstream effects of a two-signal initiation system (12, 13), including suppression of nuclear factor kappa-B (NF-κB) *via* Toll-like receptor 4 and Toll-like receptor 2 (14) and assembly of the inflammasome and activation of caspase-1 (15). Clinically, a case control study showed that low serum n-3 PUFA levels were connected with frequent gout flares (16). Although it is well established that most seafood, which typically contains large quantities of purines, leads to increased risk of gout (17, 18), whether the harmful effects of seafood in relation to gout may vary owing to different levels of n-3 PUFA in seafood is still unclear. According to the US Department of Agriculture (USDA) Dietary Research Nutrition Database, the seafood component is divided into: seafood that are high in n-3 PUFA and seafood that are low in n-3 PUFA. Whether n-3 PUFA poor seafood or n-3 PUFA rich seafood can affect gout remained to be elucidated.

Hence, the preliminary aim of this study was to determine the relationship between n-3 PUFA poor/rich seafood consumption and gout in US adults using data from the National Health and Nutrition Examination Survey (NHANES). Our study was the first large-scale cross-sectional study evaluating n-3 PUFA poor/rich seafood consumption and gout, which could shed new light on gout management.

## Materials and methods

### Study population

Potential subjects in the present study were selected from 2007 to 2016 cycle of NHANES. NHANES is a periodic, nationally representative health study conducted by the Nation Center for Health Statistics (NCHS) at the Centers for Disease Control and Prevention (CDC) aimed at evaluating individuals’ health and nutritional status. NHANES was approved by the NCHS Ethics Review Board. All subjects provided written informed consent. All data in this study are publicly accessible at http://www.cdc.gov/nchs/nhanes/.

**FIGURE 1:**
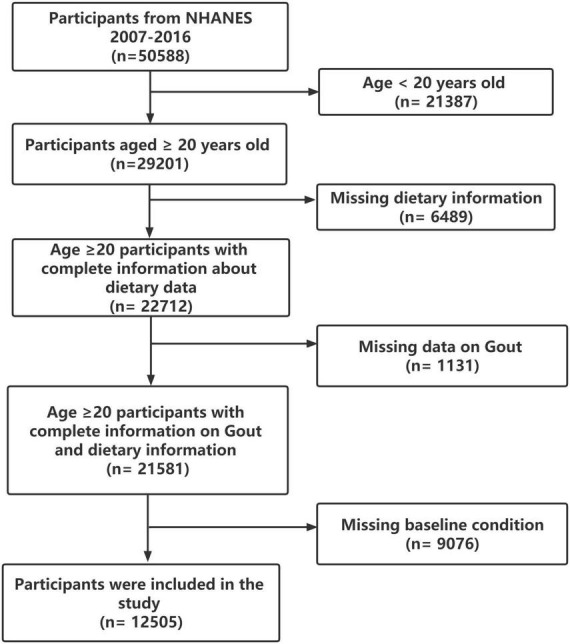
Study flowchart.

Participants aged <20 years old (*n* = 21,387), without complete information about dietary data (*n* = 6,489), missing data on gout (*n* = 1,131), or missing baseline condition (*n* = 9,076) were excluded, leaving a total of 12,505 subjects for the present analysis (Figure 1).

### Measurements and covariation assessment

#### Dietary assessment

A total of 24-h dietary recalls were utilized to calculate individual dietary intakes, which have been examined by the Nutrition Methodology Working Group (19). To ensure precise food recall and alleviate the respondent burden, eligibility criteria included subjects who had both two valid 24-h dietary recalls.

Definition of seafood was founded on the USDA Dietary Research Nutrition Database. Seafood high in n-3 PUFA included anchovy, herring, mackerel, salmon, sardine, shark, trout, and bluefin and albacore tuna. Seafood low in n-3 PUFA included catfish, clams, cod, crabs, crayfish, croaker, eel, flounder, haddock, lobster, mussels, octopus, oyster, perch, pollock, scallop, shrimp, snapper, tilapia, tuna (other than bluefin and albacore), and turtle.

#### Gout

Similar to previous NHANES reports (20), all participants were asked “Has a doctor or other health professional ever told you that you had gout?” and then classified as non-gout participants and gout participants.

### Covariables

#### Sociodemographic characteristics

According the current articles, the following variables were collected during the household interview, including age, gender, race/ethnicity, education level, and family poverty income ratio (PIR). Race/ethnicity was categorized into Mexican American, non-Hispanic Black, non-Hispanic White, other Hispanic, and other race (including multi-racial) based on NHANES classification (21). Education level was classified as less than high school, high school graduate/General Education Development (GED); some college/Associate of Arts (AA) degree and college graduate or more (22). PIR was utilized to evaluate family income. PIR was categorized into three strata (<1, 1–3, and ≥3) and defined as poor, near poor, and not poor, respectively (23).

#### Body mass index status

Body mass index (BMI) was calculated as the weight divided by the square of height (kg/m^2^). BMI <18.5 was considered as underweight, 18.5–24.9 as normal, 25–30 as overweight, and ≥30 as obese (24).

#### Smoking status

Smoking behavior was classified as never smoker, former smoker, and current smoker based on their answers to questionnaire about smoking more than 100 cigarettes in their life and whether they had quit smoking (25).

#### Drinking status

Drinking behavior was categorized as mild drinking (≤1 drink per day for women or ≤2 drinks per day for men on average over the past 12 months), moderate drinking (1–3 drinks per day for women or 2–4 drinks per day for men on average over the past 12 months), and heavy drinking (≥4 drinks per day for women or ≥5 drinks per day for men on average over the past 12 months) (26).

#### Chronic kidney disease

We used the Chronic Kidney Disease Epidemiology Collaboration formulate to assess estimated glomerular filtration rate (eGFR). Chronic kidney disease (CKD) was defined as eGFR <60 ml/min/1.73 m^2^ or urine albumin creatinine ratio (UACR) >30 mg/g (27).

#### Hypertension

Hypertension was defined as systolic blood pressure ≥140 mmHg and/or diastolic blood pressure ≥90 mmHg based on the average of three measurements of all subjects’ blood pressure (28).

#### Diabetes mellitus

Diabetes mellitus (DM) was defined as self-reported diabetes, the use of diabetes medication or insulin, glycohemoglobin (HbA1c) >6.5%, fasting glucose ≥7.0 mmol/L, random blood glucose ≥11.1 mmol/L, or 2-h oral glucose tolerance test (OGTT) blood glucose (mmol/L) ≥11.1 (29). Impaired fasting glucose (IFG) was defined as fasting plasma glucose concentration ≥5.6 and ≤6.9 mmol/L (30). Impaired glucose tolerance (IGT) was defined as a plasma glucose level ≥7.8 and ≤11.0 mmol/L at 2 h after OGTT (30).

### Statistical analysis

All statistical analyses were performed following CDC guidelines for analysis of NHANES data. A suitable sample weight was utilized to calculate all statistical analyses in order that the data corresponded to the non-institutionalized civilian population. Continuous variables were represented by mean and standard deviation (SD), whereas categorical variables were expressed by counts and weighted percentages. One-way ANOVA test (for normally distributed continuous variables), Kruskal–Wallis H-test (for non-normally distributed continuous variables) and Chi-square test (for categorical variables) were utilized to measure differences among different groups.

Weighted logistic regression analysis was set up to estimate the relationship between n-3 PUFA poor/rich seafood consumption and gout. To further examine the covariable effect on this correlation, we employed Model 1 (unadjusted), Model 2 (age, gender, and race/ethnicity were adjusted), and Model 3 (all covariates in Table 1 were adjusted). In addition, restricted cubic spline models were utilized to investigated their dose-response relationship in Model 3. Finally, we further explored the heterogenicity in different groups with interaction terms. *P* < 0.05 with effective confidence interval (CI) was of statistical significance. All analyses were constructed with R version 4.0.4^1^ (The R foundation).

**TABLE 1:** Characteristics among adults with/without gout.

Variable	Overall	Individuals without gout	Individuals with gout	*P*-value
Age, years, mean (SD)	46.29 (0.31)	45.80 (0.30)	59.50 (0.70)	<0.001
n-3 PUFA rich seafood intake, oz/day, mean (SD)	0.19 (0.01)	0.19 (0.01)	0.29 (0.06)	0.100
n-3 PUFA poor seafood intake, oz/day, mean (SD)	0.48 (0.02)	0.46 (0.02)	0.78 (0.13)	0.020
Gender, *n* (%)				<0.001
Female	5,894 (48.83)	5,777 (49.69)	117 (25.57)	
Male	6,611 (51.17)	6,189 (50.31)	422 (74.43)	
Race/ethnicity, *n* (%)				<0.001
Mexican American	1,718 (7.21)	1,687 (7.37)	31 (2.71)	
Non-Hispanic Black	2,370 (9.18)	2,240 (9.13)	130 (10.52)	
Non-Hispanic White	6,071 (73.18)	5,771 (72.95)	300 (79.17)	
Other Hispanic	1,202 (4.71)	1,169 (4.81)	33 (2.13)	
Other race (including multi-racial)	1,144 (5.73)	1,099 (5.74)	45 (5.47)	
Income, *n* (%)				0.570
Poor	2,275 (11.75)	2,178 (11.74)	97 (12.22)	
Near poor	4,841 (32.48)	4,631 (32.58)	210 (29.86)	
Not poor	5,389 (55.76)	5,157 (55.68)	232 (57.91)	
Education, *n* (%)				0.790
Less than high school	2,259 (11.64)	2,162 (11.63)	97 (11.73)	
High school graduate/GED	2,737 (20.39)	2,602 (20.34)	135 (21.88)	
Some college or AA degree	3,951 (32.94)	3,784 (32.90)	167 (33.96)	
College graduate or more	3,558 (35.03)	3,418 (35.12)	140 (32.43)	
BMI, *n* (%)				<0.001
Underweight (<18.5)	168 (35.14)	164 (1.31)	4 (0.40)	
Normal (≥18.5 and ≤24.9)	3,530 (1.28)	3,442 (30.08)	88 (13.97)	
Overweight (>24.9 and <30)	4,247 (34.08)	4,074 (34.14)	173 (32.48)	
Obese (≥30)	4,560 (29.5)	4,286 (34.47)	274 (53.15)	
CKD, *n* (%)				<0.001
No	10,706 (88.19)	10,372 (88.92)	334 (68.65)	
Yes	1,799 (11.81)	1,594 (11.08)	205 (31.35)	
DM, *n* (%)				<0.001
DM	1,858 (11.5)	1,660 (10.76)	198 (31.50)	
IFG	540 (4.5)	503 (4.31)	37 (9.47)	
IGT	507 (3.75)	478 (3.75)	29 (3.89)	
No	9,600 (80.25)	9,325 (81.18)	275 (55.13)	
Hypertension, *n* (%)				<0.001
No	7,622 (64.65)	7,514 (66.21)	108 (22.76)	
Yes	4,883 (35.35)	4,452 (33.79)	431 (77.24)	
Smoking status, *n* (%)				<0.001
Former	3,219 (26.35)	2,990 (25.70)	229 (43.73)	
Never	6,417 (52.71)	6,204 (53.13)	213 (41.34)	
Current	2,869 (20.95)	2,772 (21.17)	97 (14.92)	
Drinking status, *n* (%)				<0.001
Mild	6,166 (49.48)	5,844 (49.10)	322 (59.51)	
Moderate	2,797 (23.3)	2,710 (23.61)	87 (14.97)	
Heavy	3,542 (27.22)	3,412 (27.28)	130 (25.53)	

SD, standard deviation; n-3 PUFA, omega-3 polyunsaturated fatty acid; oz, ounce; GED, General Education Development; AA degree, Associate of Arts; BMI, body mass index; HUA, hyperuricemia; CKD, chronic kidney disease; DM, diabetes mellitus; IFG, impaired fasting glucose; IGT, impaired glucose tolerance.

## Results

### Population characteristics between groups

As illustrated in Figure 1, a total of 12,505 subjects were enrolled in this study. Table 1 summarized the population characteristics overall and between two groups (adults with/without gout). In the whole research population, the median age at baseline was 46.29 years, 48.33% were females and 51.17% were males. The overall prevalence of gout among US adults was 4.31%. Participants with gout were more likely be older, male, non-Hispanic White, obese, alcoholic, former smokers (*P* < 0.01). Additionally, they suffered from CKD, DM, hypertension, and had higher n-3 PUFA poor seafood intake (*P* < 0.05). There was no difference in income and education levels (*P* > 0.05).

### n-3 PUFA poor seafood intake is associated with higher risk of gout

**FIGURE 2:**
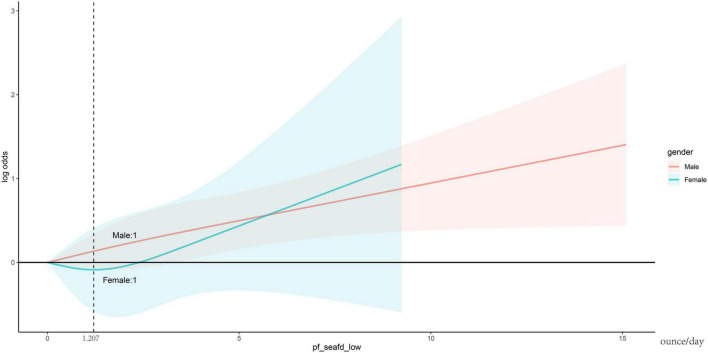
The dose-response relationship between n-3 PUFA poor seafood intake and gout. Adjusted for age, gender, race/ethnicity, income, education, BMI, CKD, DM, hypertension, smoking status, and drinking status. Solid lines hazard ratio, blue or pink area 95% confidence interval. n-3 PUFA, omega-3 polyunsaturated fatty acid; BMI, body mass index; CKD, chronic kidney disease; DM, diabetes mellitus.

**FIGURE 3:**
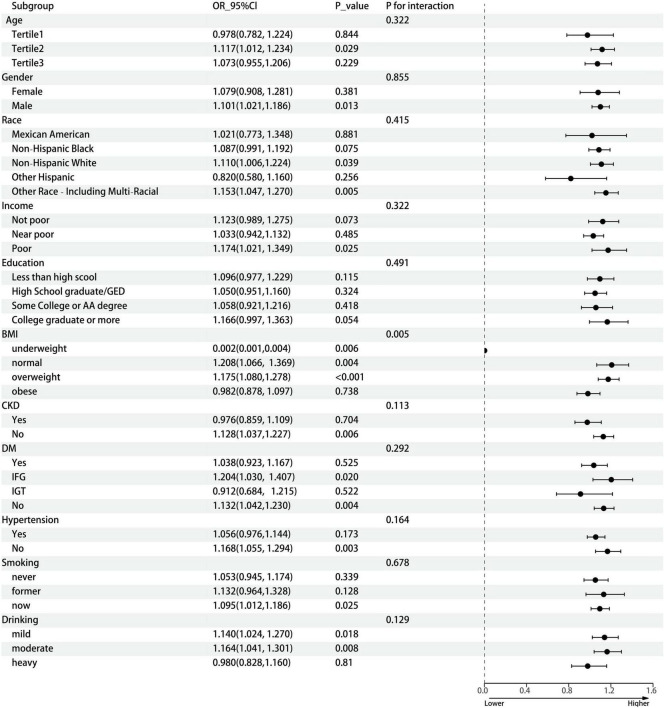
Subgroup analysis between n-3 PUFA poor seafood intake and gout. The subgroup analysis was adjusted for age, gender, race/ethnicity, income, education, BMI, CKD, DM, hypertension, smoking status, and drinking status. Age (years old), Tertile 1, 20–37, Tertile 2, 37–56, Tertile 3, 56–80; n-3 PUFA, omega-3 polyunsaturated fatty acid; OR, odds ratio; CI, confidence interval; GED, General Education Development; AA degree, Associate of Arts; BMI, body mass index; CKD, chronic kidney disease; DM, diabetes mellitus; IFG, impaired fasting glucose; IGT, impaired glucose tolerance.

Results of weighted logistic regression analysis for the association between n-3 PUFA poor/rich seafood consumption and gout are shown in Table 2. Weighted logistic regression analysis confirmed that higher n-3 PUFA poor seafood intake was associated with an increased risk of gout (Model 1, OR = 1.098, 95% CI: 1.053, 1.145, *P* < 0.001; Model 2, OR = 1.096, 95% CI: 1.048, 1.146, *P* < 0.001; Model 3, OR = 1.087, 95% CI: 1.039, 1.138, *P* < 0.001). In model 3, which adjusted for all confounding variables, the results still revealed that each unit of increase of n-3 PUFA poor seafood intake was associated with an 8.7% increased risk of gout. With respect to n-3 PUFA rich seafood intake, the connection with gout was only significant in model 1(OR = 1.112, 95% CI: 1.017, 1.216, *P* = 0.019), but become non-significant after stepwise adjusting for confounding variables (Models 2 and 3). In dose-response relationships, n-3 PUFA rich seafood consumption was non-linearly related with gout in the female group. We found an inverted L-shaped association. The prevalence of gout reached a plateau when the n-3 PUFA poor seafood intake was lower than 1.207 ounce/day in the female group. Figure 2 depicts the dose response relationship. We also conducted subgroup analyses stratified by age, gender, race/ethnicity, income, education, BMI, CKD, DM, hypertension, smoking, and drinking status to evaluate the association between n-3 PUFA rich seafood intake and gout (Figure 3). This association was similar among participants aged >37 and <56 years old. Relatively stronger relationships were also observed among male, non-Hispanic White, other race and patients with underweight, normal weight, overweight, IFG, non-DM, mild drinking, and moderate drinking. Additionally, statistically significant ORs were only present in people without CKD, hypertension and with a lower household PIR and current smoking. Besides, we also explored the heterogenicity among each subgroup with interaction terms and no significant difference was revealed among age, gender, race/ethnicity, income, education, CKD, DM, hypertension, smoking status, and drinking status (*P* for interaction >0.05 for all), revealing that the magnitude of this relationship was the same for the subjects separated into different subgroups.

**TABLE 2:** Association between n-3 PUFA rich/poor seafood intake and gout.

Variable	OR (95% CI), *P*-value		
	Model 1	Model 2	Model 3
n-3 PUFA rich seafood intake	1.112 (1.017, 1.216), 0.019	1.045 (0.947, 1.153), 0.380	1.070 (0.969, 1.183), 0.180
n-3 PUFA poor seafood intake	1.098 (1.053, 1.145), <0.001	1.096 (1.048, 1.146), <0.001	1.087 (1.039, 1.138), <0.001

Model 1: no adjustment.

Model 2: adjusted for age, gender, and race/ethnicity.

Model 3: adjusted for age, gender, race/ethnicity, income, education, BMI, CKD, DM, hypertension, smoking status, and drinking status.

OR, odds ratio; CI, confidence interval; n-3 PUFA, omega-3 polyunsaturated fatty acid; BMI, body mass index; CKD, chronic kidney disease; DM, diabetes mellitus.

## Discussion

In this nationwide cross-sectional study with 12,505 adults, a significant positive connection of n-3 PUFA poor seafood intake with gout was uncovered, revealing that higher consumption of n-3 PUFA poor seafood may contribute to an increased risk of gout. This connection remained statistically significant after we adjusted for all confounders, including age, gender, race/ethnicity, income, education, BMI, CKD, DM, hypertension, smoking status, and drinking status. Correlated subgroup analyses stratified by different variables revealed that this positive connection was not influenced, suggesting that this connection could be appropriated for different population.

It was worth noting that there was a lack of research on the effect of n-3 PUFA poor/rich seafood consumption on gout. It is well established that purine-rich foods consumption, which comprised most seafood, brings about an increased risk of gout (17). Interestingly, our study demonstrated that only n-3 PUFA poor seafood consumption was associated with increased risk of gout with statistical significance. The results of our study also provided a proof-of-concept regarding the potential for n-3 PUFA rich seafood to counteract harmful effects of purines in relation to gout. Emerging evidence has shown that n-3 PUFAs could exert protective anti-inflammatory effects. A meta-analysis revealed that supplementation of n-3 PUFAs in adults can improve inflammatory biomarkers, such as serum C-reactive protein, tumor necrosis factor α (TNF-α), and interleukin 6 (IL-6) (31).

Several hypotheses could be put forward to explain the anti-inflammatory effects of n-3 PUFAs. First, n-3 PUFAs may involve in the regulation of immunological and inflammatory responses *via* the gut microbiome (32–34). Gut microbiota can influence the metabolism and absorption of n-3 PUFAs, in the meantime, n-3 PUFAs can also affect the diversity and abundance of the gut microbiome. For example, n-3 PUFAs possibly suppressed the *Firmicutes/Bacteroidetes* ratio and the levels of *Coprococcus* and *Faecalibacterium* and increased the abundance of butyrate-producing bacterial genera, thus further reducing the inflammatory processes (35–37).

Second, n-3 PUFAs are important mediators in membrane phospholipid fatty acid composition of inflammatory cells (38). n-3 PUFAs could vary the composition of membrane phospholipid fatty acid *via* a variety of general mechanisms, resulting in affecting inflammatory cell function. To begin with, the physical properties of the membrane, such as membrane fluidity and raft structure could be altered by n-3 PUFAs (38). Then, n-3 PUFAs could exert influences on cell signaling pathways. Certain n-3 PUFAs have been shown to have anti-inflammatory effects through inhibition of the principal inflammatory cytokine *via* extracellular inflammatory stimuli (39) and G-protein coupled receptors (40). An *in vivo* experiment revealed that peroxisome proliferator-activated receptor γ, an anti-inflammatory factor, was induced by n-3 PUFAs in dendritic cells (41). Furthermore, n-3 PUFAs could alter the production in the pattern of the lipid mediators. Animal studies have shown that n-3 PUFAs may modify the fatty acid of eicosanoid, a key mediators of inflammation, resulting in producing several anti-inflammatory and inflammation resolving mediators, such as resolvins and protectins (39). Finally, with respect to T cell signaling, n-3 PUFAs were found to disturb membrane-cytoskeletal structure and function in CD4+ T lymphocytes (33).

Third, n-3 PUFAs may exert anti-inflammatory effects in macrophages. The balance between pro- and anti-inflammation is coordinated by macrophages. Previous animal studies have demonstrated that adding n-3 PUFA to the diet induced a decrease in macrophages (40, 42). In studies of mechanisms, the anti-inflammatory function by n-3 PUFAs has been proved through the free fatty acid receptor 4 protein in macrophages resulting in suppressing activity of the NF-κB complex (42). Otherwise, *in vivo* findings have been supported that n-3 PUFAs regulated inflammatory signaling in macrophages *via* the autophagic receptor SQSTM1/p62-bodies and NFE2L2 (43).

The major strengths of this population-based study are using a nationally representative sample, which facilitates the finding to be universal to a broader population. Nevertheless, several limitations cannot be ignored. First, there were only two 24-h dietary recalls in NHANES rather than three 24-h dietary recalls (two weekdays and one Friday), where recall bias is inevitable.

To minimize the influence of two-time recall bias, the NHANES design utilized sampling weight and multiple-pass method to ensure precise of dietary intake and subjects who had both two valid 24-h dietary recalls were included in this study. Second, we were unable to consider more purine-rich foods except alcohol consumption, which may lead to confounding bias. Third, cross-section design cannot draw causal relationship.

## Conclusion

In summary, our results suggest that n-3 PUFA poor seafood consumption is associated with higher risk of gout, whereas n-3 PUFA rich seafood is not and also provide a proof-of-concept regarding the potential for n-3 PUFA rich seafood to counteract harmful effects of purines in relation to gout. A dose-response analysis showed that there was a non-linear relationship between n-3 PUFA rich seafood intake and the risk of gout in the female group. Given this cross-section design, more well-designed prospective studies are warranted to validate the causal relationship between n-3 PUFA poor/rich seafood and gout.

## Data availability statement

The original contributions presented in this study are included in the article/supplementary material, further inquiries can be directed to the corresponding author.

## Author contributions

GZ and JW: conceptualization. GZ and DY: methodology. GZ, DY, and JW: software. GZ, LY, and JW: formal analysis. GZ, DY, LY, HS, JL, YW, and ZJ: data collection. GZ: writing—original draft preparation. JW: writing—review and editing and supervision. All authors read and agreed to the published version of the manuscript.

## Footnotes

1
http://www.R-project.org
